# Mixed-methods research of motivational processes in workers’ adoption of healthy behavior

**DOI:** 10.1186/s12889-024-18081-0

**Published:** 2024-02-21

**Authors:** Kayoko Ishii, Hiroko Sumita, Hitomi Nagamine, Kumiko Morita

**Affiliations:** 1 Tokyo Medical and Dental University (TMDU), 1-5-45 Yushima Bunkyo-ku, Tokyo, 113-8519 Japan; 2 Wayo Women’s University, 2-1-18 Kohnodai Ichikawa, Chiba, 272-0827 Japan

**Keywords:** Occupational health, Behavioral change, Health behavior, Lifestyle, Motivation

## Abstract

**Background:**

In occupational health, the maintenance and promotion of workers’ health, especially lifestyle motivation-based interventions, have gained considerable attention and are actively implemented. Motivational theories include self-determination theory, and some studies focus on healthy lifestyles. However, the effectiveness of health promotion interventions varies depending on the health awareness and motivation of the participants. Therefore, this study aimed to clarify the processes by which workers are motivated to improve their health and to identify the need for and type of support according to their motivation.

**Methods:**

Using a mixed-research design, an initial questionnaire survey of 94 employees (mean age = 40.97 ± 9.65) at a multicenter company in Japan, followed by semi-structured interviews with 16 employees (mean age = 40.13 ± 9.45) from the high- and low-motivation groups, were conducted. Multiple regression analysis followed by modified grounded theory-based analysis of the results of the first stage was used and the quantitative and qualitative results were integrated.

**Results:**

In the first stage, autonomous motivation scores were predicted by the behavioral change stage and relatedness satisfaction/frustration. The second stage revealed that “the process of reflecting and managing one’s own health while receiving support and feedback for maintaining and improving health” was the motivational process of workers. Result integration revealed that motivation increased through repeatedly escaping and adjusting to real problems and situational coping until the behavioral change. Despite interruptions during behavioral change, receiving feedback from others could increase motivation and continued behavioral change.

**Conclusion:**

Regardless of their level of motivation for health behaviors, workers indicated that support from others was essential. The nature of this support was found to range from providing information to offering feedback. Interventions individualized by the identified process could enable customized motivation-driven health guidance.

**Supplementary Information:**

The online version contains supplementary material available at 10.1186/s12889-024-18081-0.

## Background

The need for workplace health promotion is well recognized in many countries [1, 2], and there are many government and private award programs aimed at improving workplace health and well-being [3–5]. In the 1990s, Robert H. Rosen proposed “the healthy company” in the USA, which identified employee health management as an important management issue and suggested that companies could improve their performance, including productivity, by promoting and maintaining the health of individual employees [6]. In addition, companies that actively manage health outperform the average large US company [7]. As noted above, managing the health of employees is a priority for companies because of its impact on business performance.

In addition, many countries in the world are aging, and the proportion of the total population aged 65 and over (the aging rate) is projected to increase from 5.1% in 1950 to 9.3% in 2020 and further to 17.8% in 2060 [8]. Japan, in particular, has an aging population, and health professionals, the private sector, and the government are called upon to promote investment in health, improve the vitality of the working-age population, and extend healthy life expectancy to build a lifelong working society [9]. In Japan, only 14.9 and 9.1% of men and women, respectively, consume alcohol in amounts that increase the risk of lifestyle-related diseases. Additionally, the percentage of people with a smoking habit is 16.7%, and the smoking rate is declining annually [10]. Therefore, efforts are focused on preventing lifestyle-related diseases and managing diet and physical activity [9]. Lifestyle-related diseases account for a third of Japan’s medical costs [11] and are seen as an issue that should be addressed by the younger generation [12]. The World Health Organization states that an important way to tackle noncommunicable diseases is to reduce modifiable behavioral risk factors such as tobacco use, physical inactivity, unhealthy diet, and harmful use of alcohol [13]. In Japan, special preventive health check-ups for insured adults and their dependents (aged 40 years and older) were launched in 2008, mainly through workplaces and communities, to prevent lifestyle-related diseases such as dyslipidemia, hypertension, and diabetes [14]. Furthermore, specialized staff, including public health nurses, provide health guidance, which is specific guidance to review lifestyle individually to those who can benefit from lifestyle-related disease prevention through lifestyle improvement [15]. In addition, health management is being implemented strategically from a corporate management perspective, with reference to the USA, to maintain and promote the health of employees [16]. In this context, workplace prevention programs have had some effect in reducing weight and HbA1c in both men and women, as well as systolic blood pressure in women [17].

On the other hand, it has been pointed out that those who receive the recommended health counseling may have a high level of health awareness, while those who decline health counseling may have a low level of health awareness [18, 19]. In other words, the health awareness of those who receive health advice varies. Therefore, the challenge is how to determine the purpose, content, and goals of health counseling [13]. It is also said that in order to get them to engage in health promotion activities, it is necessary to assess each stage of behavior change and tailor it to each individual’s motivation [20]. Therefore, an intervention developed according to the health awareness and motivation for health improvement of the target population receiving health guidance is important to constitute a turning point toward healthy behavior.

The self-determination theory proposed by Deci and Ryan [21] constitutes a theory of motivation for healthy behavior from the “extrinsic motivation” stage, wherein an individual considers action based on the influence of others, to the “intrinsic motivation” stage, wherein the action is self-initiated. In basic psychological needs theory, a sub-theory of self-determination theory, three needs are important in promoting motivation: autonomy, competence, and relatedness [21]. The need for autonomy is the desire to act spontaneously and according to one’s own thoughts, the need for competence is the desire to perceive one’s competence in relation to the environment, and the need for relatedness is the desire to relate to and feel connected with intimate and significant others [22–24]. According to Ryan et al., when healthcare professionals support patients’ basic psychological needs and self-perceptions of autonomous motivation and competence, patients are more likely to experience higher levels of psychological and physical well-being [24]. Physical activity intervention programs targeting young people to support their basic psychological needs have also been shown to motivate young people to engage in physical activity [25]. On the other hand, early dropout from the program by subjects with low motivation has been identified as a challenge [26, 27]. Therefore, it is necessary to clarify how to engage subjects according to their motivation in order to induce and maintain healthy behaviors.

### Objectives

This study aimed to identify the processes that motivate workers to adopt healthy behaviors and the support they need. The first quantitative phase of the study identifies the relationship between employees’ autonomous motivation for diet and exercise and their healthy behaviors. In the second qualitative phase, based on the results of the quantitative phase, the study focuses on identifying the processes that promote autonomous motivation for diet and exercise and to identify the need for and types of support.

## Methods

### Research design

**Fig. 1:**
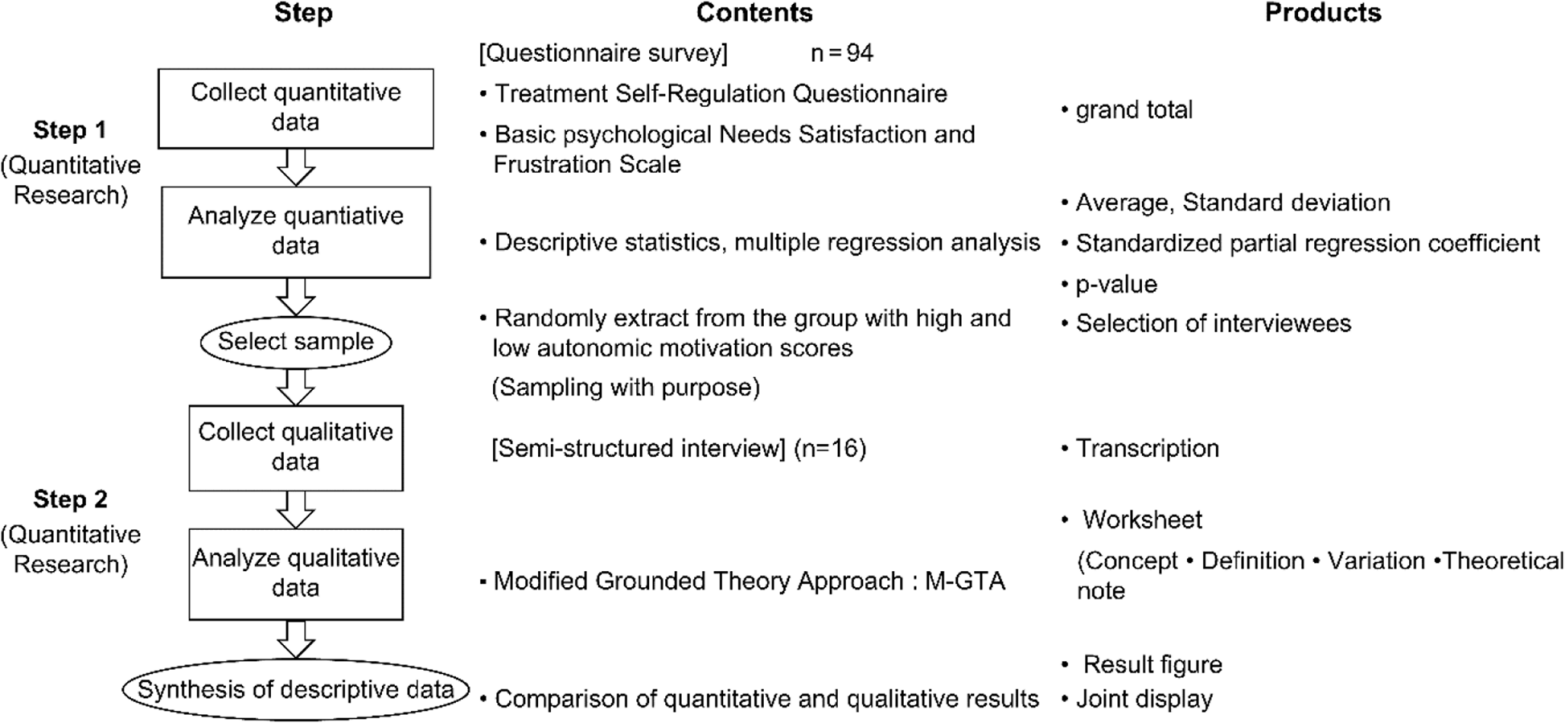
Schematic representation of the study procedures

This study involved a “descriptive, sequential design” in a mixed-research methodology, wherein quantitative data from the first stage were used to select participants for a subsequent qualitative study [28]. In the first stage involving the questionnaire survey, the related instructions were presented on the website, and participants indicated their consent through a checkmark in a check box. For the interview survey in the second stage, participants provided written informed consent. In the first stage, quantitative research data were collected and analyzed; in the second stage, the results were used to conduct a qualitative study (Fig. 1). This study was approved by the ethics committee of the Faculty of Medicine, Tokyo Medical and Dental University (M2022–027). Deidentified data, based on the ID number instead of the participant’s name, were obtained for the study analysis.

### Data collection and analysis

#### Quantitative stage

A questionnaire was presented to all 110 employees of a manufacturing company with six branches in Japan and its head office in Tokyo. Participants were aged 18–65 years, the typical Japanese working age; they lived in Japan and had regular employment. The survey was conducted from September to December 2022. The exclusion criteria included a significant psychiatric illness that might interfere with completing the study-related procedures and an inability to converse in Japanese. For the questionnaire survey, we used G Power3., set the effect size to 0.3, α-error to 0.05, and power to 0.8, which required a sample size of at least 82 respondents. Information on age, sex, occupation, and years of service were obtained as attribute-related items at the baseline. The participants completed an online self-administered questionnaire, which comprised questions from the Treatment Self-Regulation Questionnaire (TSRQ) and Basic Psychological Needs Satisfaction and Frustration Scale (BPNSFS) that are based on the stages of behavioral change in the transtheoretical model and the self-determination theory. In addition, reminder emails and a call from internal occupational health staff were sent 2 weeks before the survey deadline and the day before the collection date.

### Measures

#### Dependent variable

##### Treatment self-regulation questionnaire

The Japanese version of the TSRQ was used to measure motivation in the diet and exercise categories. Permission to use this scale for academic purposes is listed on the Self-Determination Theory website, which maintains the TSRQ scale. Additionally, Ishii et al. [29] developed a Japanese version that targets workers; Cronbach’s alphas were 0.85 for diet and 0.84 for exercise, and its reliability and validity have been confirmed. The TSRQ scale comprises 15 items each for diet and exercise and has a four-factor structure (autonomous motivation, introjected regulation, external regulation, and amotivation). The TSRQ scale includes the following: Below are 15 reasons for starting a healthy diet. On a 7-point scale, please indicate the extent to which each reason applies to you. On a 7-point Likert scale (1: not at all true to 7: very true), please indicate how applicable each reason is to you. Cronbach’s alphas for the scale in this study were 0.85 for diet and 0.84 for exercise. The TSRQ is a four-factor construct consisting of autonomous motivation, internal adjustment, external adjustment, and nonmotivation, but only autonomous motivation was extracted as a dependent variable in the present study. The reason was that although the overall Cronbach’s alpha in the current study was good, the Cronbach’s alpha for nonmotivation was 0.34 for diet and 0.65 for exercise, which were not reliable. In Japan, where the study was conducted, it was noted that some of the items related to nonmotivation are considered to be external or incorporation adjustments and are perceived differently than in the West [29]. Therefore, in the present study, Cronbach’s alpha was 0.90 for diet and 0.88 for exercise, and reliable autonomous motivation was set as the dependent variable.

#### Explanatory variables

##### Stage of behavioral change

The transtheoretical model (TTM) developed by Prochaska et al. reached Japan in the late 1990s [30]. Research reports of Japanese participants published since 2000 have shown that the higher the stage of behavior change (maintenance and implementation), the higher the breakfast consumption and the better the dietary balance [31]. There are research reports on exercise items and a scale for maintaining balanced decision-making (κ-coefficient 0.75) [32]. The behavioral change processes are expressed in the five stages of pre-contemplation, contemplation, preparation, execution, and maintenance. The scale consists of five levels, ranging from ‘I do not currently exercise and do not intend to exercise in the future’ (indifference) to ‘I currently exercise and have done so for at least 6 months’ (maintenance). Respondents were asked to select which of the five stages they fit into, ranging from ‘I currently exercise and have done so for more than 6 months’ to ‘I currently exercise and have done so for more than 6 months’.

##### Basic psychological needs satisfaction and frustration scale

The Japanese version of the BPNSFS developed by Nishimura et al. [33] was used to measure basic psychological needs. The BPNSFS measures autonomy, competence, and relatedness needs with regard to both satisfaction and frustration. The internal consistency of each need satisfaction was 0.77 for autonomy, 0.72 for competence, and 0.74 for relatedness. The internal consistency of each need frustration was 0.75 for autonomy, 0.71 for competence, and 0.78 for relatedness. In addition, the internal consistency was 0.82 for total need satisfaction and 0.83 for total need frustration [33]. The scale comprises 24 items and has a six-factor structure (autonomy satisfaction, autonomy frustration, relatedness satisfaction, relatedness frustration, competence satisfaction, and competence frustration). The scale is composed of responses to questions such as ‘I feel a sense of choice and freedom in the things I undertake’, where you can choose to indicate the degree to which the statement is true for you at this point in your life, and all items are evaluated on a 5-point Likert scale (1: completely disagree to 5: completely agree). The internal consistency of each need satisfaction was 0.80 for autonomy, 0.84 for competence, and 0.71 for relatedness. The internal consistency of each need frustration was 0.80 for autonomy, 0.77 for competence, and 0.80 for relatedness. In addition, the internal consistency was 0.84 for total need satisfaction and 0.87 for total need frustration in this study.

#### Analysis

For the questionnaire survey, we confirmed the reliability coefficient (Cronbach’s α) of each usage scale. Multiple regression analysis was performed using the stepwise method, with the TSRQ autonomy motivation score as the target variable, the six BPNSFS variables (autonomy satisfaction, autonomy frustration, relatedness satisfaction, relatedness frustration, competence satisfaction, and competence frustration) and the stage of behavior change as explanatory variables, and attributes (gender, age) as adjustment variables. SPSS V. 24 (IBM Corp., Armonk, NY, USA) was used for the analysis.

### Qualitative stage

#### Participant selection

Theoretical sampling was conducted according to the motivational score. Theoretical data saturation was targeted while comparing each concept extracted during data analysis with the theoretical notes. Finally, using theoretical sampling, 16 participants were selected. The groupings were also made with reference to the average autonomous motivation score (maximum score: 7 points) in the survey [29] of approximately 900 Japanese workers (5.6 points). The high-autonomous and low-autonomous motivation groups included participants with scores of ≥5.6 and ≤ 5.5 points, respectively. Then, individuals from each group were randomly selected as interview participants.

#### Overview of interview

We used a qualitative research approach with purposeful sampling to collect data through semi-structured interviews with participants selected in the first stage. The interview guide explored the relationship between perceptions of basic psychological needs and motivation for healthy behavior based on previous research [34] and comprised questions based on the results of the quantitative stage. Interviews were conducted online or face-to-face in a room within the company, with privacy assured, during the working hours of the interviewees and lasted 20–45 minutes. They were conducted by one researcher trained in interview methods, and they were voice-recorded and transcribed.

#### Analysis

We used the modified grounded theory approach (M-GTA) — a qualitative research approach — for the analysis. M-GTA functions by generating concepts, the smallest units that constitute a theory, from the data; examining the relationships between concepts; generating categories; and producing the analytical results. As it structures human interactions, M-GTA is suitable for generating practical theories of human behavior and comprises a model of human behavior from the analytical perspective, which evinces the analysis theme — a dynamic theory that can explain the “movements” of the analytical focus rather than chronological changes [35]. The M-GTA was considered suitable for the present study as it facilitates focusing on workers to clarify their motivational processes through interactions between workers and occupational health staff.

First, we set the analysis theme and analysis focus. Next, we generated concepts from the verbatim record using an analysis worksheet that contained four columns: column name, definition, variations (examples), and theoretical notes. For continuous comparative analysis, we investigated similar and contrasting cases and conducted the analysis. After generating multiple concepts, we investigated the relationship between concepts, repeatedly selected concepts, clarified their directions, and started generating concept-specific categories. We depicted the relationship between the concepts and categories, formulated core categories, and repeatedly examined whether these categories were appropriate. The analysis was judged complete based on the degree of completeness of concept generation by the analytical worksheets (small theoretical saturation), level of outcome diagrams, and storyline levels centered on relationships between categories (large theoretical saturation) [35].

To ensure the reliability of the research, we implemented the procedure from the formulation of the research theme to the data analysis under the supervision of a professor experienced in M-GTA implementation. Additionally, the following methods were implemented to avoid analytical bias; the accuracy of concept naming and definitions was ensured by consulting PhD students (public health and health promotion) and experts (public health, health promotion, and M-GTA).

Finally, all participants agreed to verify the suitability and explanatory power of the final theoretical model. All accepted it, and no amendments were proposed. This study’s analysis theme was “motivational process for healthy behavior in occupational health,” and the workers constituted the analysis focus.

### Integration of quantitative and qualitative results

After collecting and analyzing qualitative data, we compared the results of the multiple regression analysis of quantitative data with the categories, subcategories, concepts, and processes obtained from the semi-structured interviews. In the final analysis-and-interpretation stage, the arguments at the quantitative and qualitative stages were examined among researchers to discern convergence.

## Results

### Quantitative research phase

#### Participants

Of the 110 potential participants, 94 consented and responded to the questionnaire survey (valid response rate: 85.5%). The demographics of the participants (77 males and 17 females) are summarized in Table 1. Participant characteristics were as follows: average age: 40.97 (SD = 9.65) years; average number of years worked: 11.31 (SD = 8.305) years.

**Table 1:** Quantitative research phase participants

		TSRQ				Stage of behavioral change				BPNSF											
Variables	n (%)	Diet		Exercise		Diet		Exercise		Autonomy satisfaction		Autonomy frustration		Competence satisfaction		Competence frustration		Relatedness satisfaction		Relatedness frustration	
		Mean (SD)	t/f	Mean (SD)	t/f	Median [IQR]	z/h	Median [IQR]	z/h	Median [IQR]	z/h	Median [IQR]	z/h	Median [IQR]	z/h	Median [IQR]	z/h	Median [IQR]	z/h	Median [IQR]	z/h
Gender			-0.34^a^		0.96^a^		0.98^c^		0.11^c^		0.82^c^		0.97^c^		−1.51^c^		0.78^c^		0.91^c^		-0.10^c^
Male	77 (81.9%)	5.31 (1.34)		5.42 (1.29)		3.00 [3.00, 3.00]		3.00 [2.50, 5.00]		3.25 [3.00, 4.00]		2.50 [2.00, 3.00]		3.03 [2.75, 3.50]		2.50 [2.00, 3.00]		3.50 [3.00, 4.00]		2.00 [1.50, 2.50]	
Female	17 (18.1%)	5.43 (1.49)		5.08 (1.37)		3.00 [3.00, 4.50]		3.00 [2.50, 5.00]		3.75 [3.00, 4.25		3.00 [2.25, 3.25]		3.00 [2.25, 3.13]		2.50 [2.13, 3.25]		3.75 [3.50, 4.25]		1.75 [1.50, 2.63]	
Job position			1.21^b^		2.23^b^		14.38^d^*		11.86^d^*		4.46^d^		1.99^d^		7.76^d^		4.99^d^		7.95^d^		7.88^d^
Clerical	26 (27.7%)	5.42 (1.36)		5.45 (1.21)		3.00 [3.00, 3.25]		3.00 [3.00, 5.00]		3.50 [3.00, 4.25]		2.63 [2.19, 3.25]		3.00 [2.25, 3.25]		2.63 [2.00, 3.06]		3.63 [3.00, 4.25]		2.00 [1.50, 2.75]	
Technical	34 (36.2%)	5.10 (1.45)		5.06 (1.52)		3.00 [3.00, 3.25]		3.00 [2.75, 4.00]		3.25 [3.00, 4.00]		2.25 [1.94, 3.00]		3.00 [2.75, 3.50]		2.50 [2.00, 3.00]		3.50 [3.00, 4.00]		2.00 [1.44, 2.31]	
Manufacturing	12 (12.8%)	4.94 (1.43)		4.93 (0.95)		2.00 [1.00, 3.00]		2.00 [1.00, 3.00]		3.00 [2.50, 3.94]		2.88 [2.00, 3.38]		2.50 [2.06, 3.56]		2.75 [2.13, 3.19]		3.38 [2.69, 3.94]		2.25 [1.75, 2.94]	
Sales	18 (19.1%)	5.79 (1.03)		5.87 (0.99)		3.00 [3.00–5.00]		3.00 [3.00, 5.00]		3.75 [3.19, 3.81]		2.50 [2.19, 2.81]		3.25 [2.75, 3.56]		2.13 [2.00, 2.75]		3.75 [2.69, 4.06]		2.25 [1.69, 2.50]	
Others	4 (4.3%)	Only 4 participants in this group																			
Living with somebody			3.13^b^		2.72^b^		1.76^d^		0.01^d^		0.15^d^		4.51^d^		0.26^d^		0.55^d^		5.17^d^		2.16^d^
Living alone	11 (11.7%)	4.85 (1.42)		4.76 (1.47)		3.00 [2.50, 3.00]		3.00 [2.50, 5.00]		3.25 [3.00, 4.25]		2.25 [1.75, 3.00]		3.00 [2.63, 3.38]		2.25 [2.00, 3.00]		3.25 [2.63, 3.75]		2.00 [1.50, 2.88]	
Living with partner	14 (14.9%)	5.90 (0.83)		5.82 (0.99)		3.00 [3.00, 5.00]		3.00 [3.00, 3.50]		3.63 [3.00, 4.00]		3.00 [2.00, 3.31]		3.00 [2.44, 3.25]		2.25 [1.81, 2.88]		4.00 [3.19, 4.50]		1.50 [1.25, 2.06]	
Living with family (Child, and/or parents)	69 (73.4%)	5.90 (0.83)		5.82 (0.99)		3.00 [3.00, 5.00]		3.00 [3.00, 3.50]		3.63 [3.00, 4.00]		3.00 [2.00, 3.31]		3.00 [2.43, 3.25]		2.25 [1.81, 2.88]		4.00 [3.19, 4.50]		1.50 [1.25, 2.06]	
Work Place			−1.48^a^		-1.12^a^		2.50^c^*		2.39^c^*		0.96^c^		-1.01^c^		1.05^c^		−0.69^c^		0.71^c^		−0.04^c^
Main office	54 (57.4%)	5.54 (1.31)		5.51 (1.36)		3.00 [3.00, 3.50]		3.00 [3.00, 5.00]		3.50 [3.00, 4.00]		2.50 [2.00, 3.00]		3.00 [2.75, 3.50]		2.50 [2.00, 3.00]		3.75 [3.00, 4.38]		2.00 [1.50, 2.50]	
Branch office	40 (42.6%)	5.09 (1.40)		5.18 (1.22)		3.00 [2.00, 3.00]		3.00 [2.00, 4.50]		3.25 [2.81, 4.00]		2.63 [2.06, 3.00]		3.00 [2.31, 3.50]		2.50 [2.00, 3.00]		3.50 [3.06, 4.00]		2.00 [1.31, 2.50]	

SD: Standard Deviation, IQR: Interquartile Range

*p <  0.05. ^a^Student’s t-test; ^b^One Way analysis of variance with Bonferroni correlation; ^c^Mann–Whitney U test; ^d^Kruskal–Wallis test with Bonferroni correction

Table 1 shows that manufacturing workers had the lowest behavior change (diet h = 14.38, *p* <  0.05; exercise h = 11.86, *p* <  0.05). Table 2 shows the results of the correlation analysis. There was a negative correlation between gender and job position (r = − 0.490, *p* <  0.001). Therefore, only gender was included in the final regression model, as occupation was not significant after controlling for other variables.

**Table 2:** Results of Peason correlation test for factors associated with TSRQ

Variables	1	2		3		4		5	6	
1)TSRQ(Diet)										
2)TSRQ(Exercise)										
3)Age	0.207		0.213							
4)Gender	0.036	–	0.100		0.002					
5)Job Position	0.091		0.146		0.019	–	0.490***			
6)Living with somebody	0.052		0.095	–	0.115	–	0.214	0.158		
7)Work Place	0.153		0.116	–	0.043	–	0.043	0.081	–	0.162

***p < 0.001

### Multiple regression analysis

The results of multiple regression analysis using the stepwise method were as follows. The predictors of autonomous motivation for diet in linear regression analysis (R^2^ = 0.353) and for exercise (R^2^ = 0.459) are presented in Table 3. Stage of behavior change for diet (β = 0.351, *p* <  0.001), relationship satisfaction (β = 0.373, *p* <  0.001), and relationship frustration (β = 0.201, *p* = 0.047) were positive predictors of autonomous motivation. Stage of behavior change for exercise (β = 0.396, *p* <  0.001), relatedness satisfaction (β = 0.428, *p* <  0.001), and relatedness frustration (β = 0.193, *p* = 0.034) were also positive predictors of autonomous motivation.

**Table 3:** Predictors of autonomous motivation according to linear regression analysis

Outcome	Autonomy(Diet)^a^							Autonomy(Exercise)^b^						
Variables	β		B		SE	*p*	VIF	β		B		SE	*p*	VIF
Age	0.168			0.024	0.304	0.054	1.015	–	0.136	–	0.461	0.266	0.097	1.010
Gender	–	0.030	–	0.106	0.012	0.729	1.014		0.133		0.018	0.011	0.087	1.018
Stage of behavioral change		0.351		0.429	0.112	< 0.001	1.145		0.396		0.412	0.088	< 0.001	1.156
BPNSF(Relatedness satisfaction)		0.373		0.666	0.183	< 0.001	1.428		0.428		0.732	0.162	< 0.001	1.461
BPNSF(Relatedness frustration)		0.201		0.379	0.189	0.047	1.356		0.193		0.350	0.163	0.034	1.318
R^2^		0.353							0.459					

The Shapiro-Wilk test was used to test for normal distribution of the residuals, *p* = 0.42 for diet and *p* = 0.07 for exercise

The Breusch-Pagan test was used to test for heteroscedasticity, *p* = 0.1328 for diet and *p* = 0.1350 for exercise

^a ^F = 9.606; Durbin–Watson value = 2.158; ^b^ F = 14.911; Durbin–Watson value = 2.034

*VIF* variance inflation factor

Based on the abovementioned results, the explanatory variables that affected the autonomous motivation score were the stage of behavioral change and relatedness satisfaction and frustration, which accounted for 35% of the diet item and 46% of the exercise item.

### Qualitative research phase

#### Participants

In the qualitative stage, participants were selected based on the results of the initial quantitative stage. A total of 18 people were recruited, of whom only 16 were interviewed because one person did not give consent and one person reported that he could understand and speak plain Japanese, but could not satisfactorily explain his views on health in a language other than his mother tongue. In the current study, it was considered that there were three groups of people: those with high motivation to diet and exercise, those with low motivation to diet and exercise, and those with high motivation to either diet or exercise and low motivation to both. This grouping was therefore chosen. The results showed: seven people with a high autonomous motivation score for diet and exercise, six with a low autonomous motivation score for diet and exercise, and three with high and low scores each for diet or exercise. The characteristics of the participants (14 men and 2 women; average age 40.13 [SD = 9.45] years) are shown in Table 4.

**Table 4:** Qualitative research phase participant characteristics (*n* = 16)

Participant	Gender	Age	Job Position	Living with somebody	Work Place	TSRQ^a^ (Diet)	TSRQ ^a^ (Exercise)
1	Male	20–29	Technical	Family member	Main	High	High
2	Male	20–29	Sales	Living alone	Main	High	High
3	Female	30–39	Clerical	Family member	Main	High	High
4	Male	30–39	Technical	Family member	Main	High	High
5	Male	30–39	Technical	Family member	Main	High	High
6	Male	30–39	Manufacturing	Living alone	Main	Low	Low
7	Male	30–39	Manufacturing	Family member	Branch	Low	Low
8	Male	30–39	Sales	Family member	Main	High	High
9	Male	40–49	Technical	Family member	Branch	High	High
10	Female	40–49	Manufacturing	Family member	Branch	High	Low
11	Male	40–49	Manufacturing	Family member	Branch	Low	Low
12	Male	40–49	Manufacturing	Family member	Branch	Low	Low
13	Male	40–49	Sales	Family member	Main	High	High
14	Male	50–59	Technical	Family member	Main	High	High
15	Male	50–59	Manufacturing	Family member	Branch	Low	Low
16	Male	60–69	Technical	Partner only	Main	High	High

^a^The TSRQ was scored on a seven-point scale, with scores of 5.6 and above in the high group and 5.5 and below in the low group

### M-GTA

Qualitative results yielded two related behavioral skill categories (Thinking about behavior and Behavior) and a Self-management skills category; six subcategories (Information provision, Autonomy, Feedback for correction/enhancement, Interruption, Escaping and adapting to reality, Foundation); and 18 concepts.

### Storyline

**Fig. 2:**
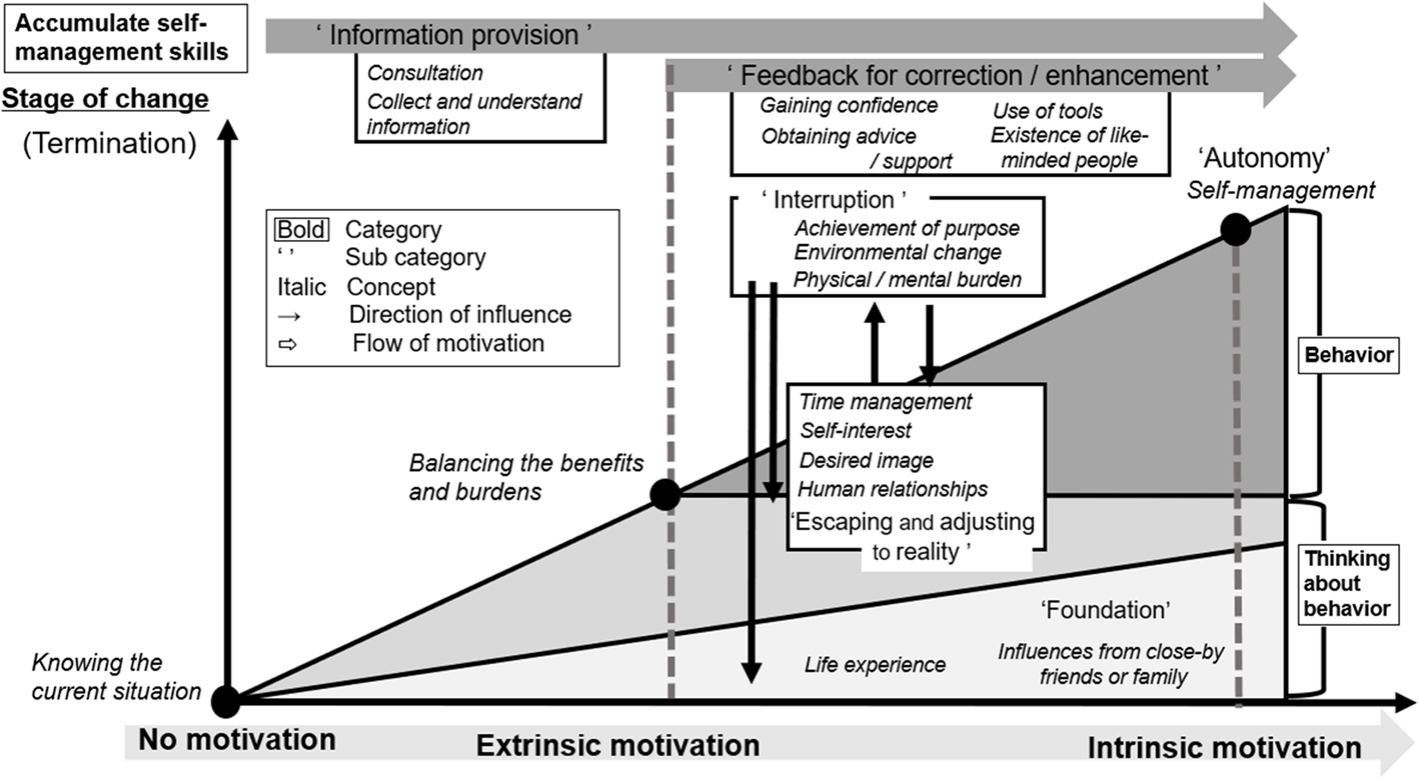
Schematic representation of the outcome

The categories, subcategories, and concepts are denoted by terms within boldface, quotation marks, and italic face, respectively. Workers construct themselves with *life experiences* and *influences from close people* as a ‘foundation.’ By *knowing the current situation* with these fundamental values and ways of thinking, the workers then deepen their thinking about behavior. At this time, workers *collect information* about health, deepen their understanding, and sometimes *consult.* While reflecting on their behavior, the workers deepen their awareness of *time management*, *self-interest*, *desired image*, and *human relationships*, *balancing the benefits and burdens* while repeatedly ‘escaping and adjusting to reality.’ Afterward, the workers transition to behavior, wherein some workers face something that serves as an ‘interruption’ of a behavior, such as *achievement of purpose*, *environmental change*, or *physical/mental burden*, following which they would return to the stage that was before the implementation of that behavior or of knowing the current situation. However, as a result of *gaining confidence* by experiencing change, and of ‘corrective and reinforcing feedback’ such as the *existence of like-minded people*, *advice and support* from surrounding people, and the *use of tools*, the workers can accumulate self-management skills for promoting motivation toward health. As shown here, *self-management* becomes possible when motivation is promoted. Even if there is an escape or interruption in life, this can be handled, or adjustments can be made by the workers themselves, and lead to maintenance and improvement of health (Fig. 2).

For the analysis theme of “motivational process for healthy behavior in occupational health,” we identified a “process of self-reflection and management of one’s own health while receiving support and feedback to maintain and promote health.”

### Synthesis phase

In Table 5, we describe the content of the categories in the motivational process, including the differences between the high-autonomic and low-autonomic motivation score groups, with the definition of the concepts. Table 6 presents a joint display that integrates the quantitative and qualitative results.

**Table 5:**
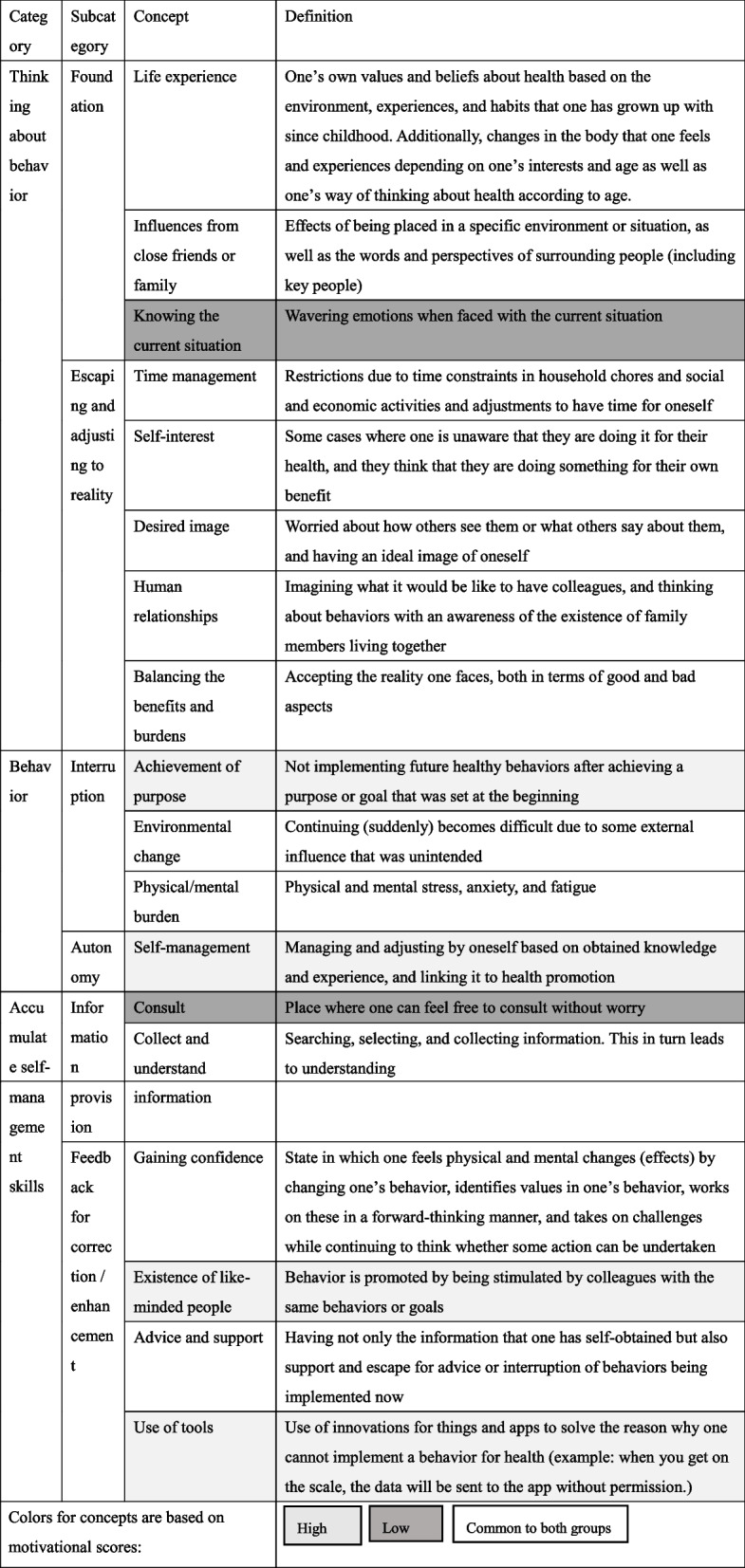
Concepts and categories of motivation for workers’ healthy behaviors’

**Table 6:** Joint display

Quantitative research results	Quantitative follow-up interview	How do qualitative research results explain the quantitative research results?
Autonomous motivation score is low when the behavioral change stage and relatedness satisfaction score are low and the relationship frustration score is high.	・There are times when one is faced with the current situation and there are wavering emotions; however, no behavior has been implemented. ・Although one thinks about implementing a behavior, one tries to move away from the change due to time constraints and human relationships. ・Information provision and consultation are needed.	It became clear that before the behavior changed, the worker repeatedly escaped from the problems they faced in reality and made adjustments accordingly. It became clear that the motivation increased upon coming to terms with reality.
Autonomous motivation score is high when the behavioral change stage and relatedness satisfaction score are high and the relationship frustration score is low.	・While accepting the reality and implementing behaviors, one is faced with an event that once again interrupts the behavior. ・By receiving external feedback, the behavior can be resumed and continued.	It was clarified that the worker faced reasons for interruption even when continuing the behavior; however, motivation increased because of feedback from others, and allowed continued behavioral change.

## Discussion

In the present study, we determined that the variables related to workers’ motivation regarding healthy behavior included variables in the behavioral change stage and levels of satisfaction or frustration. A wide range of workers involved in occupational health participated in this study, including clerical, technical, manufacturing, and sales workers.

### Explanatory variables for motivation

The multiple regression analysis in the first stage showed that the motivation for diet and exercise could be predicted by the behavioral change stage and relatedness satisfaction/frustration. According to self-determination theory, the factors that promote motivation are the satisfaction of basic psychological needs such as autonomy, competence, and relatedness, and the absence of need barriers [24]. A study by Pedersen et al., designed to support the satisfaction of basic psychological needs related to PA, found that increased awareness that one’s basic psychological needs were supported by their colleagues likely facilitated major improvements in motivation [27]. Thus, the presence of others, such as colleagues, and their mutual relationships, are important in promoting motivation. Meanwhile, the present study did not include autonomy and competence as explanatory variables. Nishimura [36] indicated the possibility that the concepts of being “autonomous” and “competent” in Japan differ from those in the West, depending on the country and culture. Japanese self-determination is not based on the self alone but also depends on the presence of others and the situation one is in at the time [37]. Japan is seen as a family-centered society; therefore, the autonomy that is generated by individualistic Western countries is often incompatible with issues related to medical ethics in Japan. Even in autonomy, the term “relational autonomy” is commonly used in nursing, medical care, etc. [38]. Therefore, Japanese individuals tend to make choices based on values that emphasize relationships with groups rather than individual values.

### Storyline

In the qualitative analysis in the second stage, the motivational process for healthy behavior in occupational health was clarified, and two categories related to behavior and one category related to subject skill were included. The first category related to behavior was thinking about behavior. The person was influenced by their experiences and environment from childhood, the values cultivated, and close friends and family, which became their foundation. According to Kamran et al., [39] the patients’ age showed a significant inverse correlation with self-efficacy, perceived benefits, affects related to behavior, interpersonal influences, and commitment. Thus, attributes influence different variables. Afterward, when the participant sees the results of medical examinations or other assessments, they *know the current situation* and face it, thereby developing wavering emotions and deepening their thought by inferring that it may be better to implement some behavior. Interventions based on the health belief model, which increases the likelihood of people being aware of threats, balancing benefits and drawbacks, and adopting healthy behaviors, have the greatest effect on treatment adherence, including for hypertension, among young people [40]. Therefore, the awareness of threats, situational severity, and one’s own vulnerabilities is an important stage. Additionally, participants came to terms with their family and social life, imagined and made innovations regarding implementing behaviors, and repeatedly escaped and adjusted. At the stage of implementing a behavior, which is the second category, there were cases where one would interrupt their behavior after achieving the initial purpose or due to a change of the environment wherein the behavior was implemented or physical and mental burdens. The behavior will likely be continued while balancing the benefits and burdens.

For the accumulation of self-management skills, which is a category pertaining to subject skill, it was clarified that information provision and consultation were important before implementing a behavior. According to Hazavehei et al., [41] in populations with low knowledge scores on healthy fruit and vegetable consumption behavior, over half of the participants were in the indifference and interest periods of the behavioral change stages, and fruit and vegetable consumption was also low, with a clear correlation between both aspects. Therefore, knowledge acquisition is important, and support is also necessary. Furthermore, it is important to have relationships and support that allow participants to feel changes in their behavior and improve their self-confidence. According to Deci et al., meaningful changes occur when one accepts oneself, becomes curious about the reasons for one’s behavior, and decides to do things differently [34]. Even if there is an escape or interruption in one’s behavior in one’s daily life, relationships with others enable one to find meaning in one’s own behavior and address the issue or make adjustments.

### Synthesis

In the present study, we clarified the elements of the process whereby motivation is promoted. In the first quantitative stage, the basic psychological needs of autonomy and competence were not included in the variables that predict motivation. However, in the second qualitative stage, self-management, adjustment, and self-confidence, similar to autonomy and competence, emerged. Therefore, in Japan and other regions identified via meta-analysis, fulfilling the three basic psychological needs likely increases motivation toward health, as indicated in previous research [42].

At the stage when motivation is not cultivated, it is important to discern the participant’s view of health and their background. It is also necessary to support them in understanding the current situation. According to Gorin et al., [43] people with high autonomy support experienced more weight loss than those without such support. Chatzisarantis et al. showed that autonomy support motivates physical activity and is important from the early stages of behavioral change [25]. Later, in the stage before implementing the behavior, the participant faces a barrier, such as lack of time due to balancing family and social life, which is unique to the working population. In such cases, it is important to clarify the factors that make it difficult for the participant to take action and to present resources that can be used. Additionally, continuous autonomy support is necessary to ensure the participant can deepen their reason for adopting healthy behaviors.

Even when the implementation of a behavior is interrupted, it is important in health guidance to provide support to deepen thinking about the behavior and to provide feedback on the resultant changes and actions toward the healthy behavior. According to previous research, messages about progress toward goals and reminder messages about activities conferred a sense of accomplishment and self-confidence [44].

Based on the above-described facts, in health guidance, consistent provision of appropriate information, conveying a supportive presence, and giving feedback are important to strengthen and correct behaviors after the participant begins to implement behavioral change. Thus, it may be possible to increase motivation for healthy behaviors by intervening while utilizing elements stratified by the degree of motivation. Although basic psychological needs such as autonomy and competence were not significant as variables in the first stage of multiple regression analysis, the concept of autonomy and the development of self-confidence were extracted in the second qualitative stage, which also suggested the need for autonomy and competence. As basic psychological needs are universal across race, country, and culture [45], the motivational processes identified in this study may be valid outside the country in which the study was conducted. Based on the stage of behavioral change, this study has identified methods to motivate health behaviors, and the usefulness of the intervention program should be tested in the future by implementing the program in the target population.

### Limitations of the study

The following four limitations of the study need to be noted. First, because this was a cross-sectional study, caution should be exercised in interpreting causal relationships. Second, because this study focused on a single company in the Japanese manufacturing industry, there are limitations in the generalizability of the findings to groups with different social, economic, and cultural backgrounds. In the future, it will be necessary to conduct surveys with more subjects from other industries, with different working conditions, and with different characteristics to achieve generalizability. Third, the data obtained from the questionnaires in the first stage and the interviews in the second stage were subjective. This study clarified a methodological approach according to the degree of motivation for behavioral change toward health, and it was impossible to clarify whether this was related to actual health. To overcome this limitation, it is important to conduct further research that includes the results of blood sampling, medical history, medication history, hospital visit history, etc., which indicate the objective health status of the participants. Finally, the M-GTA used in the qualitative analysis is an analytical method that clarifies interactions. In this study, interviews were conducted only with workers covered by occupational health services. Therefore, further research is important, as it is necessary to collect data from the staff providing occupational health services and to study the interaction between workers and occupational health staff descriptively in the future.

## Conclusion

In this study, we clarified that the variables related to the motivation for workers’ healthy behavior included the behavioral change stage and relatedness satisfaction or frustration. The motivation between the objective and explanatory variables was promoted through a “process of reflection and management of one’s own health with support and feedback to maintain and improve health.” Furthermore, from the relationship perspective, despite the diverse content and manner of involvement depending on the degree of motivation, the presence of others was consistently indispensable. Thus, we identified that individualized health guidance can be provided by implementing interventions developed according to the degree of motivation.

### Supplementary Information

**Supplementary material 1.**

## Data Availability

The datasets used and/or analyzed during the current study can be made available by the corresponding author on reasonable request.

## Abbreviations

TSRQ: Treatment self-regulation questionnaire
BPNSFS: Basic psychological needs satisfaction and frustration scale
TTM: Transtheoretical model
M-GTA: Modified grounded theory approach
