# Supplementary material for: Mixed-methods research of motivational processes in workers’ adoption of healthy behavior
Source: BMC Public Health. 2024 Feb 21;24:537. doi: 10.1186/s12889-024-18081-0 (PMC10880196; doi:10.1186/s12889-024-18081-0)
Supplement: Supplementary file 1 — Supplementary material 1. [file 12889_2024_18081_MOESM1_ESM.docx]

| How do you view eating to maintain or improve your health? |
| --- |
| Why do you think so? |
| Have you ever changed your thoughts on food or your eating habits? |
| What triggered the change? |
| Are you currently taking any actions for your health regarding your diet? |
| Do you have any restrictions or goals for your diet? |
| Have you ever experienced a connection between your actions and desired results? |
| Have you ever shared your views on health, awareness, etc. with anyone? |
| Have people around you ever said anything about your view of your health or your actions for health? |
| What kind of support would you feel would enhance your health support? Or, what kind of support would you feel your health was enhanced with? |

**Appendix**

Interview Guide (excerpts)
